# MARK2 regulates C9orf72 repeat–associated non-AUG translation

**DOI:** 10.1073/pnas.2514182122

**Published:** 2025-11-13

**Authors:** Yu-Ning Lu, Xiangning Li, Lindsey Hayes, Xiao-Feng Zhao, Jiou Wang

**Affiliations:** ^a^Department of Biochemistry and Molecular Biology, Bloomberg School of Public Health, Johns Hopkins University, Baltimore, MD 21205; ^b^Brain Science Institute and Department of Neurology, Johns Hopkins University School of Medicine, Baltimore, MD 21205; ^c^Department of Neuroscience, School of Medicine, Johns Hopkins University, Baltimore, MD 21205

**Keywords:** integrated stress response, eIF2α, MARK2

## Abstract

Protein homeostasis is exquisitely regulated through processes involving protein synthesis essential for cellular health and disease prevention. Repeat-associated non-AUG (RAN) translation at expanded GGGGCC repeats in the *C9orf72* gene produces dipeptide repeat (DPR) proteins that are implicated in amyotrophic lateral sclerosis and frontotemporal dementia (C9-ALS/FTD). However, the mechanisms promoting this noncanonical translation remain incompletely understood. Here, we identify microtubule affinity-regulating kinase 2 (MARK2) as a key eIF2α kinase that enhances RAN translation under proteotoxic stress. We show that MARK2-eIF2α signaling, activated by misfolded proteins including DPRs and TDP-43, is upregulated in C9-ALS patient tissues. Loss of MARK2 significantly suppresses RAN translation in reporter cells, patient-derived neurons, and a mouse model and confers neuroprotection under proteotoxic conditions. These findings position MARK2 as a critical stress-sensing cytosolic regulator that promotes repeat-associated noncanonical translation and associated toxicity.

Disruption of protein homeostasis is a hallmark of neurodegenerative diseases such as ALS and FTD (1, 2). The most common genetic cause, a GGGGCC repeat expansion in the *C9orf72* gene, drives pathogenesis through multiple mechanisms, including RAN translation that produces cytotoxic dipeptide repeat proteins DPRs (3). RAN translation is facilitated by phosphorylation of eIF2α, a key event in the integrated stress response (ISR), typically mediated by kinases such as PKR, PERK, HRI, and GCN2 (4). We recently identified MARK2 as an eIF2α kinase activated by cytosolic proteotoxic stress (5). However, its role in RAN translation remains unclear. Here, we demonstrate that MARK2-eIF2α signaling is activated by neurodegeneration-associated protein misfolding stress, including DPRs. MARK2 is essential for promoting RAN translation, and its knockdown reduces DPR production. These findings reveal a previously unrecognized mechanism regulating RAN translation and highlight MARK2 as a potential therapeutic target in ALS/FTD.

## Results and Discussion

**Fig. 1.**
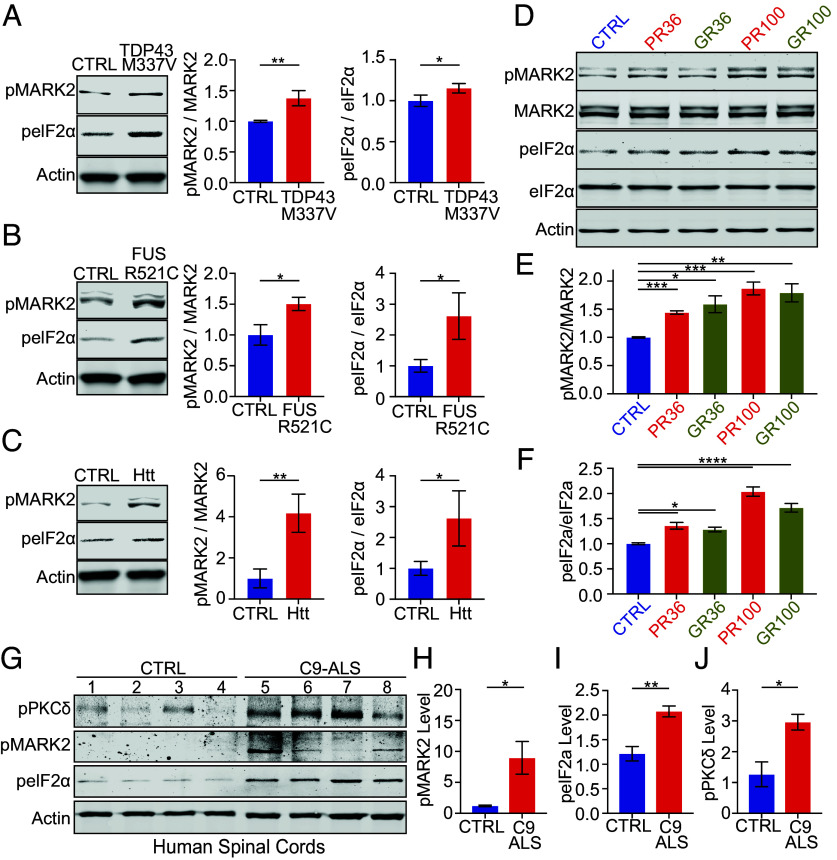
MARK2-eIF2α signaling responds to cytosolic proteotoxic stress. (*A*–*C*) Immunoblot analysis of HEK293 cells transfected with TDP-43^M337V^, Htt, or FUS^R521C^ to assess the phosphorylation of MARK2 and eIF2α (n = 3). (*D*–*F*) Immunoblot analysis of MARK2 and eIF2α phosphorylation in HEK293 cells expressing poly-GR (GR36, GR100) or poly-PR (PR36, PR100) (n = 4). (*G*–*J*) Representative immunoblots of PKCδ, MARK2, and eIF2α in spinal cord tissues from C9HRE-ALS patients and non-ALS controls (C9HRE-ALS: n = 4; CTRL: n = 4).

The ISR promotes eIF2α phosphorylation to limit global translation and induce stress-responsive gene expression. To determine whether MARK2, a direct cytosolic kinase for eIF2α, responds broadly to proteotoxic stimuli associated with neurodegenerative diseases, we examined mutant proteins linked to neurodegenerative diseases. In HEK293 cells, expression of ALS/FTD-associated mutants TDP-43^M337V^ or FUS^R521C^ (6–8) induced strong phosphorylation of MARK2 and eIF2α, indicating activation of this pathway (Fig. 1 *A* and *B*). A similar increase was observed with polyglutamine-expanded huntingtin (Htt) associated with Huntington’s disease (9), supporting MARK2 as a general sensor of cytosolic proteotoxicity (Fig. 1*C*). The hexanucleotide repeat expansion in *C9orf72* linked to ALS/FTD undergoes RAN translation via bypassing AUG start codon and produces DPRs that are intrinsically toxic and may act as proteotoxic stressors (3). Expression of glycine-arginine or proline-arginine (GR36/PR36) DPRs increased phosphorylation of MARK2 and eIF2α, with the longer GR100/PR100 repeats exhibiting a stronger effect (Fig. 1 *D*–*F*), indicating that DPRs activate MARK2 signaling in a length-dependent manner.

To assess disease relevance, we examined postmortem spinal cord tissues from C9-ALS patients. Compared to control, C9-ALS samples displayed elevated phosphorylation of PKCδ (Fig. 1 *G* and *J*), MARK2 (Fig. 1 *G* and *H*), and eIF2α (Fig. 1 *G* and *I*), consistent with PKCδ acting as the sensor for protein misfolding stress and upstream activator of MARK2 signaling (5). These findings suggest that the PKCδ–MARK2–eIF2α axis is engaged in C9-ALS pathogenesis and may be driven by proteotoxic stress from DPRs and other misfolded proteins.

**Fig. 2.**
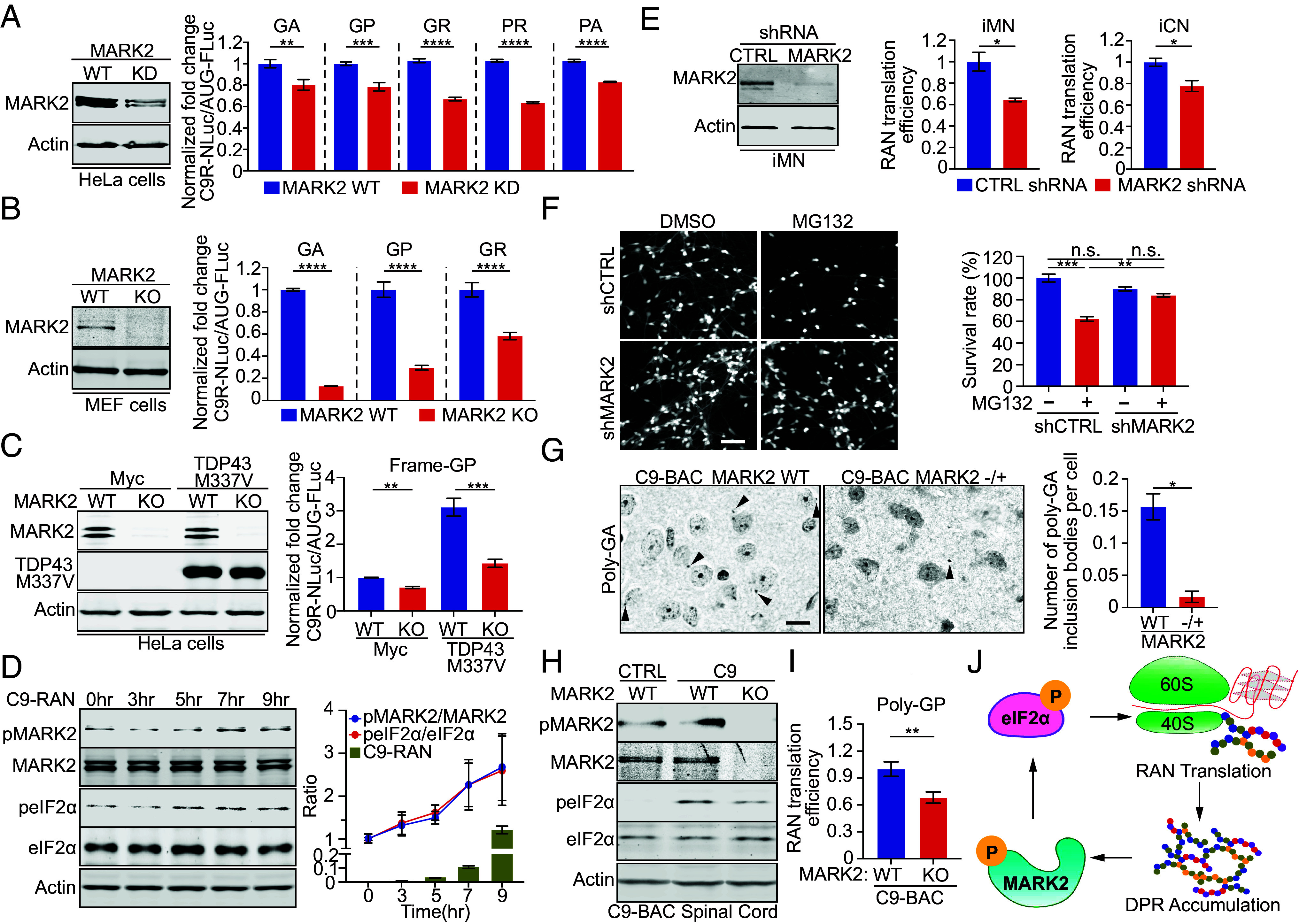
Lack of MARK2 expression reduces the C9-RAN translation in patient-derived neurons and mice. (*A*) HeLa Flp-In cells were subjected to MARK2 knockdown (shRNA or CRISPR gRNAs), followed by induction of RAN translation and analysis. Immunoblot analysis confirmed MARK2 reduction, and the bar graphs represent RAN translation quantification (GA n = 9, GP n = 8, GR n = 4, PR n =4, and PA n =4). (*B*) Wild-type (WT) and MARK2 knockout (KO) MEF cells were transfected with the RAN translation reporter plasmid, and the RAN translation was analyzed. Immunoblot analysis confirmed MARK2 reduction, and the bar graphs represent RAN translation quantification (GA n = 3 and GP n = 3, and GR n =12). (*C*) HeLa Flp-In cells depleted of MARK2 via CRISPR gRNAs and expressing TDP-43^M337V^ were analyzed for RAN translation. Immunoblot analysis confirmed TDP-43^M337V^ and MARK2 levels. The bar graphs represent RAN translation quantification (n = 3). (*D*) HeLa Flp-In cells induced for RAN translation over varying durations were analyzed for MARK2 and eIF2α phosphorylation. Line and the bar graphs show quantification of MARK2/eIF2α phosphorylation and RAN translation (n = 3). (*E*) iPSC-iMNs and iCNs were subjected to MARK2 knockdown using shRNA, and poly-GP was analyzed using ELISA. Immunoblot analysis confirmed reduction of MARK2, and the bar graphs represent ELISA quantification (n = 3). (*F*) Viability of iCNs was measured by Calcein-AM staining after MARK2 knockdown and MG132 treatment (0.25 μM, 48 h). The bar graphs represent viability quantification (n = 3). (Scale bar, 100 μm.) (*G*) IHC staining indicates that reduction of MARK2 decreased poly-GA inclusions in C9-BAC mouse brain tissues. For each group, 4 to 6 images from three mice were analyzed. (Scale bar, 5 μm.) (*H*) Immunoblot analysis of spinal cord tissues from B6, C9-BAC, and C9-BAC/MARK2KO mice. The bar graphs represent immunoblot quantification (n = 3). (*I*) Quantification of RAN translation from spinal cord lysates of C9-BAC and C9-BAC/MARK2KO mice (n = 7). (*J*) Schematic of the feedback loop in which MARK2, the key cytosolic eIF2α kinase, promotes RAN translation at expanded *C9orf72* repeats.

To test whether MARK2 regulates RAN translation, we employed dual-luciferase reporter HeLa cells stably expressing (GGGGCC)_70_ repeats upstream of a non-AUG NanoLuc coding sequence in all five DPR reading frames, including poly-GA, GP, or GR (sense) (10) and poly-PR or PA (antisense) (11). Knockdown of MARK2 by shRNA or CRISPR significantly reduced RAN translation in all frames, without affecting canonical Fluc translation (Fig. 2*A*). Further, MARK2-deficient MEFs showed even greater suppression of RAN translation compared to wild-type (WT) cells (Fig. 2*B*), indicating that MARK2 promotes efficient RAN translation. We also examined whether proteotoxic stress promotes RAN translation through MARK2. Our results indicate that expression of TDP-43^M337V^ specifically increases eIF2α phosphorylation (Fig. 1*A*), and in reporter cells, TDP-43^M337V^ expression further elevated RAN translation (Fig. 2*C*). To examine the temporal relationship between RAN translation and MARK2-eIF2α signaling, we performed time-course experiments. Induction of RAN translation for increasing durations resulted in a corresponding rise in MARK2 and eIF2α phosphorylation (Fig. 2*D*), indicating that RAN translation can directly activate the MARK2-eIF2α pathway. Together, these results demonstrate a feedback loop in which MARK2-eIF2α signaling not only promotes RAN translation but is also stimulated by proteotoxic stresses including DPR production.

To evaluate the role of MARK2 in disease-relevant neurons, we performed shRNA knockdown of MARK2 in iPSC-derived motor neurons (iMNs) and cortical neurons (iCNs) from C9-ALS patients. In both neuronal types, reduction of MARK2 significantly decreased poly-GP levels, indicating suppression of RAN translation (Fig. 2*E*). Furthermore, to evaluate MARK2’s role in proteotoxic stress-induced neurotoxicity, we inhibited the proteasome using a low-dose MG132 (0.25 μM) treatment that did not induce significant neuronal death in WT neurons but caused marked death in C9-ALS iCNs. Importantly, MARK2 knockdown improved survival under MG132 stress (Fig. 2*F*), indicating that suppressing MARK2 signaling protects neurons from proteotoxic stress.

We next evaluated MARK2 in vivo using C9-BAC transgenic mice, which express the human *C9orf72* gene with ~500 repeats (12). Despite its lack of behavioral phenotypes, the mice provide an in vivo model for evaluating RAN translation (13). Compared to WT mice, C9-BAC spinal cords exhibited elevated MARK2 and eIF2α phosphorylation, recapitulating findings from patient tissues (Fig. 2*H*). Crossing with MARK2 knockout (KO) mice abrogated this phosphorylation and markedly reduced poly-GP levels in ELISA analysis (Fig. 2*I*). Immunohistochemistry further revealed reduced poly-GA inclusions in brain tissues with MARK2 deficiency (Fig. 2*G*). These results establish MARK2 as a critical upstream regulator of RAN translation in the mammalian CNS and support its role in DPR pathogenesis.

Our study identifies MARK2 as a key eIF2α kinase that promotes RAN translation at expanded *C9orf72* repeats. MARK2 broadly responds to proteotoxic stress from misfolded DPRs and TDP-43, with activity correlating with eIF2α phosphorylation and elevated DPR levels in cells, neurons, and tissues from C9-ALS patients and mice. This establishes a self-amplifying feedback mechanism wherein RAN-derived DPRs and other disease-associated proteotoxic stresses activate MARK2 signaling, which in turn enhances DPR production (Fig. 2*J*). Unlike previously known ISR kinases such as PKR or PERK, which are activated by double-stranded RNA or endoplasmic reticulum stress, respectively, and have been reported to influence RAN translation (10, 14, 15), MARK2 uniquely senses cytosolic misfolded proteins, representing a distinct regulatory branch of the stress response. Targeting MARK2 with small molecules or RNA-based strategies may provide a therapeutic approach to disrupt the self-amplifying cycle of RAN translation and proteotoxicity in C9-ALS/FTD.

## Methods

RAN translation was analyzed by reporters or ELISA in HeLa, MEF, and hiPSC-derived neurons. Tissues from C9-BAC mice were subjected to IHC, ELISA, and western blotting analyses. Detailed *Methods* are provided in the *SI Appendix*.

## Supplementary Material

Appendix 01 (PDF)

## Data Availability

All study data are included in the article and/or *SI Appendix*.
