# Supplementary material for: MARK2 regulates C9orf72 repeat–associated non-AUG translation
Source: Proc Natl Acad Sci U S A. 2025 Nov 13;122(46):e2514182122. doi: 10.1073/pnas.2514182122 (PMC12646204; doi:10.1073/pnas.2514182122)
Supplement: Supplementary file 1 — Appendix 01 (PDF) [file pnas.2514182122.sapp.pdf]

PNAS

Supplemental Information for

MARK2 Regulates C9orf72 Repeat-associated Non-AUG Translation

Yu-Ning Lu[a], Xiangning Li[a], Lindsey Hayes[b], Xiao-Feng Zhao[a], and Jiou Wang[a,c,1]

[1] To whom correspondence should be addressed:

Jiou Wang, Johns Hopkins University, 615 N. Wolfe Street, E8410, Baltimore, MD 21205 USA,

Email: jiouw@jhmi.edu

This file includes:

Supplemental text

Methods and Materials

DNA plasmids

For mammalian expression, the TDP-43^{M337V} has been subcloned into the pLenti CMV Puro DEST (W118-1, Addgene) plasmid as previously described (1); the FUS^{R521C} has been subcloned into the mammalian expression vector, pRK5-myc, plasmid as previously described (2); the polyQ82 expression vector (mHTT-N171-82Q) was described previously (3). The Flag-tagged constructs, including PR36 and PR100, were each subcloned in a mammalian expression vector, pcDNA3.1-CMV-3×nFlag vector, using a Gateway Single-Fragment BP/LR reaction.

Mammalian Cell lines, transfections, and drug treatments

Mouse embryonic fibroblasts (MEFs), HeLa Flp-In cells, and human embryonic kidney 293 (HEK293) cells (ATCC, CRL-3216) were grown in Dulbecco's modified Eagle's medium (DMEM) supplemented with 10 % fetal bovine serum (FBS) and antibiotic-antimycotic solution at 37 °C with 5 % CO_2, including MARK2 WT and knockout MEFs (4). MARK2 knockout in HeLa was achieved by infecting cells with viruses derived from pLenti-CRISPR v2 harboring the MARK2-specific gRNA (5'-TGGCAGGATGAAAGAAAAAG-3'), and a population of puromycin-selected cells were used. Transfection of mammalian cells was performed using Lipofectamine 2000 (Invitrogen). Briefly, 2 µg of the DNA plasmids and 4 µl of Lipofectamine 2000 were mixed in 500 µl Opti-MEM I (Invitrogen) and applied to HEK293 cells in 2 ml DMEM supplemented with 10 % FBS. After two days post-transfection, cells were lysed for analysis. MG132 was dissolved at 20 mM in dimethylsulfoxide (DMSO), and Doxycycline was dissolved at 20 mg/ml in DMSO. All drugs were diluted in DMEM/F12 before the cell treatments. MARK2 knockdown in HeLa, iMN, and iCN was achieved by infecting cells with viruses derived from pLKO-1 harboring the MARK2

shRNAs (TRCN0000001581, TRCN0000001583, and TRCN0000001584, Dharmacon) and a population of puromycin-selected cells ware used.

CRISPR-Cas9 gene editing

The MARK2-specific gRNA sequences (5'-TGGCAGGATGAAAGAAAAAG-3') were selected by using the CRISPR design tool from Benchling, Inc. The gRNAs were cloned into the gRNA/Cas9-expressing vector pLenti-CRISPR v2, conferring resistance to puromycin (Addgene 52961). The resulting cell lines were verified for probing the targeted protein through immunoblot analysis.

iPSC culture, motor neuron (iMN) differentiation, and cortical neuron (iCN) differentiation

Human iPSCs were obtained from the National Institute of Neurological Disorders and Stroke, and all samples were fully de-identified prior to receipt, in accordance with institutional and ethical guidelines. Human iPSC culturing and motor neuron differentiation were performed as previously described (5). The iPSCs were cultured in StemFlex medium (Thermo Fisher Scientific, A3349401) on plates coated with Matrigel (Corning, 354230). To generate motor neurons, iPSCs were first differentiated into neuroepithelial progenitor (NEP) cells using neural medium [1:1 DMEM/F12:neurobasal medium, GlutaMAX Supplement (Thermo Fisher Scientific, 35050061), N-2 supplement (Thermo Fisher Scientific, 17502048), B-27 supplement (Thermo Fisher Scientific, 17504044), and ascorbic acid], supplemented with 3 µM CHIR99021, 2 µM SB431542, and 2 µM DMH-1 for 6 d. NEP cells were then split into Matrigel-coated plates and grown in neural medium supplemented with 1 µM CHIR99021, 2 µM SB431542, 2 µM DMH-1, 0.1 µM retinoic acid (RA), and 0.5 µM purmorphamine to generate motor neuron progenitor (MNP) cells. To generate motor neuron-like cells, MNPs were dissociated by cell scraper and

placed in suspension culture using neural medium supplemented with 0.5 μM RA and 0.1 μM purmorphamine. After 8 d, motor neuron-like cells were dissociated and plated onto PDL (MilliporeSigma, P7405)/Laminin (Corning, 354259)-coated plates. Mature motor neurons were generated in 12 d using neural medium supplemented with 0.5 μM RA, 0.1 μM purmorphamine, and 0.1 μM compound E, with medium change every other day before experimental analysis.

The method used for converting the human iPSCs into cortical neurons was based on a previous report with minor modifications (6). Briefly, on day 1, the medium was changed to neural induction medium [1:1 DMEM/F12:neurobasal medium, GlutaMAX Supplement, N-2 supplement, B-27 supplement, NEAA supplement (MilliporeSigma, TMS-001-C), Insulin-Transferrin-Selenium (Thermo Fisher Scientific, 41400045), and 2-Mercaptoethanol (MilliporeSigma, 21985-023)], supplemented with 10 μM SB431542 (Ambeed), and 0.25 μM LDN-193189 (TargetMol) for 7 d. Cells were then dissociated using Accutase (MilliporeSigma, A6964) and seeded into PDL /Laminin-coated 10-cm dishes in neural induction medium plus 10 μM SB431542 and 0.25 μM LDN-193189. On day 10, the medium were changed to neural induction medium alone for culture another 7 d. On day 17, cells were dissociated with Accutase and seeded at a density of 2×10^6 cells per well (6-well plates) or 0.8×10^6 cells per well (12-well plates) into PDL/Laminin-coated plates with neuronal maintenance medium (Neurobasal medium, GlutaMAX, and B27) supplemented with 10 μM DAPT. Cells were cultured for an additional 7 days with medium change every 2 days before experimental analysis.

Cell viability assay using Calcein-AM

Cell viability was assessed using live-cell staining probe Calcein-AM (Invitrogen), where fluorescence intensity corresponds to number of viable cells. After experimental treatments, neurons were seeded into a 24-well plate, infected with the TDP-43^{M337V} lentivirus for 24 h and

treated with MG132 (0.25 μM) or an equivalent volume of DMSO as a solvent control. Cells were then washed with PBS and incubated with 1 μM Calcein-AM for 30 min at 37 °C in the dark under 5 % CO_2. Fluorescence images were captured with Nikon TS100 fluorescence microscope. Additionally, a Synergy H1 hybrid reader (BioTek) with a custom filter set (485 nm excitation and 535 nm) was used to measure the Relative fluorescence intensity (RFI).

Animals and brain lysate preparation

The C9-500 transgenic mice (The Jackson Laboratory, strain 029099), MARK2 knockout mice (The Jackson Laboratory, strain 009365) (kindly provided by Dr. Helen Piwnica-Worms) (4), and control WT mice were bred at the Johns Hopkins mouse facilities. At 3 months of age, the mice were euthanized in a CO_2 chamber and the brains and spinal cords were harvested followed by flash-freeze in liquid nitrogen and stored at -80 °C for RNA and protein analysis. For spinal cord lysates, half spinal cord was sonicated in ice-cold homogenate buffer, RIPA buffer (Millipore Sigma R0278), EDTA-free protease inhibitor (Roche, USA), phosphatase inhibitor cocktail (Sigma-Aldrich P8340), phosphatase inhibitor 3 (Millipore Sigma P0044) at 1:10 wt/vol ratio until the tissue was completely dissolved. The mixture was then spun at 16,000g for 10 min and the supernatant was collected.

Immunohistochemistry staining

Paraformaldehyde-fixed mouse brain tissues were paraffin-embedded and sectioned at 5 μm thickness for poly-GA IHC-staining. Sections were deparaffinized in xylene, rehydrated through graded ethanol, and subjected to antigen retrieval using antigen retrieval buffer (Abcam, catalog ab93684) following the manufacturer’s instructions. To reduce nonspecific binding, slides were first incubated with Serum-Free Super Block (ScyTek, catalog 61886) for 10 min, then placed in

blocking buffer (10 % BSA, 0.3 % Triton X-100 in PBS) for 1 h at room temperature. Tissue sections were incubated overnight at 4 °C with anti-poly-GA antibody (Proteintech, catalog 24492-I-AP; 1:200) diluted in blocking buffer. After three PBS washes, slides were treated with 0.3 % H_2O_2 in PBS for 15 min, followed by incubation with biotinylated goat anti-rabbit IgG (H+L) (Vector Laboratories, catalog BA-1000; 1:500) for 1 h at room temperature. Sections were then incubated with ABC reagent (Vector Laboratories, catalog PK-7100) for 30 min at room temperature, and immunoreactivity was visualized using the DAB Substrate Kit (Vector Laboratories, catalog K-4100). Slides were counterstained with hematoxylin (MilliporeSigma, catalog 51275), dehydrated through graded ethanol and xylene, mounted with DPX (MilliporeSigma, catalog 06522), and sealed with clear nail polish. Images were acquired using a ZEISS microscope with a ×40 objective, focusing on dark puncta located adjacent to but outside the nucleus. Image analysis was performed manually in ImageJ (NIH, version 1.53) without applying intensity or size thresholds.

Dipeptide Repeat ELISA

For the poly-glycine/proline (GP) or poly-glycine/alanine (GA) ELISA, the supernatants from cell lysates and mouse tissue lysates were collected, and the protein concentration was determined by Pierce BCA protein assay (Thermo Fisher Scientific). According to previously established protocols (7), the samples were diluted to 1 mg/mL. 0.375 µg/mL biotinylated rabbit anti-GP antibody was incubated in 96-well small-spot streptavidin-coated plates at room temperature for 1 h. The plate was washed with PBST three times, and 35 µl of cell lysate was added in each well with duplicates and incubated at room temperature for 3 h. The plate was washed with PBST for another time and incubated in sulfo-tagged detection antibodies at a concentration of 1 µg/ml for 1 h. After further PBST washes, 150 µl of read buffer was added, and the samples

were immediately read by MESO QuickPlex SQ 120. HEK193 cell with overexpression in GFP-tagged dipeptide repeat protein was used as a dipeptide specificity control.

Western blotting

Cells were washed with PBS for twice and placed on ice. A lysis buffer was added, containing RIPA buffer (50 mM Tris-HCl (pH 7.6); 150 mM NaCl; 1 % NP-40; 1 % SDS; 100 mM sodium fluoride; 17.5 mM β-glycerophosphate; 0.5 % sodium deoxycholate; 10 % glycerol), EDTA-free protease inhibitor (Roche, USA), phosphatase inhibitor cocktail (Sigma-Aldrich), 1 μM phenylmethanesulfonyl fluoride. Lysate were kept cold on ice and pulse-sonicated, and then centrifuged at 12,000g at 4 °C for 10 min. The protein concentration of each sample was determined by bicinchoninic acid (BCA) assay (Thermo Fisher). Equal amount of each protein extract was run by SDS-PAGE and transferred to nitrocellulose membrane (Millipore HATF08550, USA). The membranes were blocked with 5 % non-fat powdered milk and 0.05 % NaN_3 in TBST. The membranes were incubated in primary antibody in 5 % w/v BSA and 0.05 % NaN_3 TBST overnight, then finally incubated with appropriate secondary antibodies. The results were imaged with Li-Cor Odyssey or Syngene GeneGenome and analyzed with Image Studio software (LiCor 9120, USA). The antibodies used include those against eIF2α (Cell Signaling 5324, USA), peIF2α (Cell Signaling 9721), Actin (Santa Cruz SC-47778), pPKCδ-^{505}T (Cell Signaling 9374), MARK2 (Abcam ab135816; Santa Cruz SC-365405), pMARK2-^{595}T (Abcam ab34751), TDP43^{M337V} (Abcam 10782-2-AP).

Virus production

Lentivirus production was carried out using HEK293T cells cultured under standard conditions. The cells were co-transfected overnight with a lentiviral transfer plasmid containing the gene of

interest, the psPAX2 packaging plasmid (Addgene #12260), and the pMD2.G envelope plasmid (Addgene #12259) using Lipofectamine 2000 (ThermoFisher #11668019). After 72 h post-transfection, the medium containing lentiviral particles was collected, filtered through a 0.45 μm PVDF membrane (Millipore Sigma HVHP02500), and mixed with PEG8000 at a 3:1 ratio. The mixture was incubated with constant rocking at 60 rpm for 4 h at 4 °C and centrifuged at 1,600 × g at 4 °C for 1 h. The supernatant was removed, and the viral pellet was resuspended in PBS, aliquoted, and stored at −80 °C.

C9orf72 RAN translation reporter assay

The C9orf72 RAN translation reporter assay was performed as previously described (8). Briefly, HeLa Flp-In cells [expressing sense reading frames GA (glycine-alanine), GP (glycine-proline), and GR (glycine-arginine), or antisense reading frames PR (proline-arginine) and PA (proline-alanine) (8, 9)] were seeded in 6-well plates at a density of 2×10^5 cells per well and transfected with MARK2 shRNA for 24 h. The following day, cells were treated with 2 μg/ml doxycycline for an additional 24 h. For MEF cells, 2×10^5 cells were plated in 6-well plates and transfected with a RAN translation luciferase-based reporter plasmid (Frame-GA, Frame-GP, or Frame-GR) for 24 h. Cells were then treated with 2 μg/ml doxycycline for 24 h. After treatment, HeLa Flp-In and MEF cells were harvested, and NLuc and FLuc luciferase activities were measured using the Nano-Glo Dual-Luciferase Reporter Assay System (Promega) on a BioTek™ Synergy™ H1 Hybrid Microplate Reader.

Statistical analysis

Statistical analyses were conducted using Student’s t-test for comparisons between the two groups and one-way ANOVA followed by Tukey post hoc test for comparisons among multiple

groups, performed with GraphPad Prism software. Uless otherwise specified, the sample size "n" refers to biological replicates. A P value <0.05 was considered statistically significant. Error bars represent ± SEM. *p ≤ 0.05; **p ≤ 0.01; ***p ≤ 0.001; ****p ≤ 0.0001.

References

1. T. Zhang, G. Baldie, G. Periz, J. Wang, RNA-processing protein TDP-43 regulates FOXO-dependent protein quality control in stress response. *PLoS Genet* **10**, e1004693 (2014).
2. T. Zhang *et al.*, FUS Regulates Activity of MicroRNA-Mediated Gene Silencing. *Mol Cell* **69**, 787-801.e788 (2018).
3. S. Subramaniam, K. M. Sixt, R. Barrow, S. H. Snyder, Rhes, a striatal specific protein, mediates mutant-huntingtin cytotoxicity. *Science* **324**, 1327-1330 (2009).
4. J. B. Hurov *et al.*, Immune system dysfunction and autoimmune disease in mice lacking Emk (Par-1) protein kinase. *Mol Cell Biol* **21**, 3206-3219 (2001).
5. Y. Liu *et al.*, A C9orf72-CARM1 axis regulates lipid metabolism under glucose starvation-induced nutrient stress. *Genes Dev* **32**, 1380-1397 (2018).
6. A. Dannert, J. Klimmt, C. Cardoso Gonçalves, D. Crusius, D. Paquet, Reproducible and scalable differentiation of highly pure cortical neurons from human induced pluripotent stem cells. *STAR Protoc* **4**, 102266 (2023).
7. H. Liu *et al.*, A Helicase Unwinds Hexanucleotide Repeat RNA G-Quadruplexes and Facilitates Repeat-Associated Non-AUG Translation. *J Am Chem Soc* **143**, 7368-7379 (2021).
8. W. Cheng *et al.*, C9ORF72 GGGGCC repeat-associated non-AUG translation is upregulated by stress through eIF2alpha phosphorylation. *Nature communications* **9**, 51 (2018).
9. W. Cheng *et al.*, CRISPR-Cas9 Screens Identify the RNA Helicase DDX3X as a Repressor of C9ORF72 (GGGGCC)n Repeat-Associated Non-AUG Translation. *Neuron* **104**, 885-898.e888 (2019).
